# Antihypertensive drugs and pancreatic cancer risk in patients with chronic pancreatitis: a Danish nationwide population-based cohort study

**DOI:** 10.1038/s41416-019-0562-y

**Published:** 2019-09-02

**Authors:** Jakob Kirkegård, Frank Viborg Mortensen, Deirdre Cronin-Fenton

**Affiliations:** 1 Department of Surgery, Section for Hepato-Pancreato-Biliary Surgery, Aarhus University Hospital, Aarhus, Denmark; 2 Department of Clinical Epidemiology, Aarhus University Hospital, Aarhus, Denmark

**Keywords:** Pancreatic cancer, Risk factors

## Abstract

**Background:**

Antihypertensives may inhibit pancreatic carcinogenesis. We examined the association between use of these drugs and pancreatic cancer in patients with chronic pancreatitis.

**Methods:**

We conducted a nationwide population-based cohort study of all chronic pancreatitis patients diagnosed in Denmark during 1996–2012. Using a Cox proportional hazards model with time-varying exposure lagged by 1 year, we examined the risk of pancreatic cancer according to antihypertensive drug use.

**Results:**

We included 8,311 patients with chronic pancreatitis and observed 153 pancreatic cancers during follow-up. At baseline, 2197 patients (26.4%) were exposed to at least one class of antihypertensive drugs. We did not observe any measurable associations between the use of antihypertensive drugs and pancreatic cancer.

**Conclusions:**

Our findings suggest little evidence of an association between the use of antihypertensive drugs and pancreatic cancer risk in patients with chronic pancreatitis. Confirmation is warranted in future studies.

## Introduction

Chronic pancreatitis is an inflammatory disease characterised by progressive and irreversible destruction of the exocrine and endocrine pancreas and may eventually progress to pancreatic cancer.^1^ Pancreatic carcinogenesis in chronic pancreatitis patients may be inhibited by antihypertensive drugs. Experimental evidence suggest that several classes of antihypertensive drugs have anticancer properties (e.g., inhibition of pancreatic stellate cells, a key player in pancreatic carcinogenesis, by drugs acting on the renin–angiotensin system and induction of pancreatic cancer cell apoptosis by beta-blockers).^2,3^ Thus, antihypertensive drugs may have multiple effects on pancreatic carcinogenesis, which could decrease the risk of pancreatic cancer in patients with chronic pancreatitis and improve survival in patients with pancreatic cancer. However, findings from epidemiological studies are ambiguous.^4–6^ One study found that the use of drugs acting on the renin–angiotensin system had limited effect on pancreatic cancer risk in healthy individuals,^4^ but it was associated with an improved prognosis in pancreatic cancer patients.^5^ Other investigators suggested that beta-blockers could improve pancreatic cancer prognosis.^6^ Given their widespread use and generally favourable risk profiles, any potential anticancer properties of antihypertensive drugs is intriguing, as these could be used as both preventive and therapeutic agents. It is particularly important to investigate if these drugs could affect pancreatic cancer risk among patients with chronic pancreatitis, as these patients have an inherently higher risk of pancreatic cancer compared with the general population.^1^ We therefore conducted a nationwide population-based cohort study to examine the potential association between the use of antihypertensive drugs and pancreatic cancer risk in patients with chronic pancreatitis.

## Methods

We have previously described the study design and analytic framework in detail.^7^ In brief, we used the Danish National Patient Registry to identify a cohort of all patients with a first-time diagnosis of chronic pancreatitis in Denmark during 1996–2012. Individual-level data linkage to the Danish Cancer Registry, Danish National Prescription Registry and the Danish Civil Registration System was used to obtain information on pancreatic cancers, comorbidities, prescription drug use and vital status. We followed patients from 1 year after their chronic pancreatitis diagnosis until pancreatic cancer, death, emigration or 31 December 2015, whichever occurred first.

We assessed the use of antihypertensive drugs (angiotensin-converting enzyme (ACE) inhibitors, aldosterone receptor antagonists, angiotensin-II receptor antagonists, beta-blockers, calcium channel blockers and diuretics), requiring at least two filled prescriptions of the same drug class to be considered exposed. We considered drug exposure to be time varying with a 1-year lag period, allowing patients to switch between exposed and unexposed status. We considered the exposure to be continuous if two prescriptions plus their days’ supply overlapped, allowing a 30-day grace period for delays in prescription filling.

For each drug class, we calculated the crude incidence rate ratio as the ratio between the incidence rate among drug users compared with non-users. Using Cox regression, we estimated the hazard ratio (HR) of pancreatic cancer comparing drug users with non-users. In the multivariable model, we adjusted for age (restricted cubic spline with three knots), sex, socioeconomic status, year of chronic pancreatitis diagnosis, Gagne Comorbidity Index score^8^ and use of other antihypertensive drugs. In a supplementary analysis, we additionally adjusted for alcohol-related and smoking-related diseases to assess potential confounding from exposure to these substances.

All estimates are presented with associated 95% confidence intervals (CIs).

## Results

We identified 8,311 patients with incident chronic pancreatitis in Denmark during the study period. Median age was 54 years (IQR: 45–64 years), and 5,498 (66.2%) were men (for full descriptive characteristics, please see Online Supplementary Information). In total, 153 pancreatic cancers were diagnosed during 60,365 person-years of follow-up (median 5.0 years, IQR: 2.3–8.9 years). The overall distribution of the risk estimates for the drug classes appeared to be centred around the null with few exceptions (Table 1). Our findings indicated that users of aldosterone receptor antagonists may have lower risk of pancreatic cancer, whereas the risk may be elevated in users of calcium channel blockers. However, these estimates were imprecise (Table 1). Findings from our supplementary analysis showed that adjustments for alcohol-related and smoking-related diseases did not have any measurable impact on our estimates.

**Table 1:** Risk of pancreatic cancer in 8,311 patients diagnosed with chronic pancreatitis in Denmark during 1996–2012 according to exposure to antihypertensive drugs

	Events	Person-years	Incidence rate^a^	Crude HR	Adjusted HR^b^
*ACE inhibitor*					
Non-users	134	52,828	2.56 (2.14–3.00)	1.00 (reference)	1.00 (reference)
Users	19	7,536	2.52 (1.61–3.95)	1.10 (0.68–1.77)	0.81 (0.49–1.33)
*Aldosterone receptor antagonist* ^c^					
Non-users	>140	58,613	2.56 (2.18–3.00)	1.00 (reference)	1.00 (reference)
Users	< 10	1,752	1.7*^c^ (0.55–5.31)	0.65 (0.21–2.02)	0.55 (0.17–1.75)
*Angiotensin-II receptor antagonist*					
Non-users	141	56,295	2.50 (2.12–2.95)	1.00 (reference)	1.00 (reference)
Users	12	4,069	2.95 (1.67–5.19)	1.39 (0.77–2.52)	0.98 (0.49–1.66)
*Beta-blocker*					
Non-users	139	54,865	2.53 (2.15–2.99)	1.00 (reference)	1.00 (reference)
Users	14	5,450	2.55 (1.51–4.30)	1.07 (0.62–1.86)	0.83 (0.47–1.47)
*Calcium channel blocker* ^c^					
Non-users	>140	58,678	2.47 (2.10–2.91)	1.00 (reference)	1.00 (reference)
Users	<10	1,686	4.7*^c^ (2.37–9.49)	1.96 (0.96–4.00)	1.56 (0.76–3.22)
*Diuretics*					
Non-users	122	54,411	2.37 (1.99–2.83)	1.00 (reference)	1.00 (reference)
Users	31	8,954	3.46 (2.43–4.92)	1.50 (1.09–2.22)	1.09 (0.71–1.69)

^a^Per 1,000 person-years

^b^Adjusted for age (restricted cubic spline with three knots), sex, socioeconomic status, year of chronic pancreatitis diagnosis, Gagne Comorbidity score and use of other antihypertensive drugs

^c^Numbers collapsed for confidentiality

*Final number collapsed for confidentiallity

## Discussion

We did not observe any clinically relevant association between use of antihypertensive drugs and pancreatic cancer risk in patients with chronic pancreatitis. Chronic pancreatitis patients have a particularly high risk of pancreatic cancer,^1^ possibly due to sustained pancreatic inflammation. These patients generally exhibit poor lifestyle choices with a high frequency of tobacco smoking and high alcohol consumption.^9^ As these substances are closely associated with pancreatic cancer, they may mitigate a potential anticancer effect of antihypertensive drugs.

Despite some important strengths including the population-based design and a high positive predictive value of chronic pancreatitis diagnoses in the Danish National Patient Registry,^10^ some limitations should be noted. Confounding by indication may have affected our estimates, as users of antihypertensive drugs may have more comorbidity than non-users and thus potentially a higher baseline risk of pancreatic cancer. However, users of antihypertensive drugs may also have better compliance with medical treatments and healthy lifestyle choices than non-users. This is supported by a lower prevalence of alcohol-related and smoking-related diseases among users of several classes of antihypertensive drugs compared with the entire population, which may mitigate the effect of higher comorbidity levels. Pancreatic cancer was a rare event, so some of the confidence intervals were wide. However, the estimates appeared to be centred around the null, limiting concerns of imprecise estimates. We had information on redeemed prescriptions, but not actual drug consumption. As compliance with prescription drugs among chronic pancreatitis patients may be impaired, some misclassification of exposure may be present. Such misclassification would be non-differential, biasing our estimates towards the null. We had limited information on consumption of alcohol and exposure to tobacco smoking, as this information was captured using proxy diagnoses. Because such diagnoses are under-recorded in the Danish National Patient Registry, residual confounding from these substances may be present. However, we have previously shown that the alcohol-related diagnoses recorded in the Danish National Patient Registry are a valid proxy for alcoholism in chronic pancreatitis, whereas the smoking-related diagnoses are not an optimal proxy.^10^ Also, our exposure definitions implied that patients with few prescriptions had equivalent exposure to patients with many prescriptions. Such an exposure definition is unable to capture a potential dose-response relationship, which should be examined in a larger study.

In summary, our study suggested little evidence of an association between the use of antihypertensive drugs and pancreatic cancer risk in patients with chronic pancreatitis. Our findings warrant confirmation in larger studies.

## Supplementary information

Supplementary Information

## Data Availability

The data underlying this paper cannot be shared without violation of Danish law. Researchers can obtain data themselves by applying at www.sundhedsdatastyrelsen.dk (Danish Health Data Authority).
