# Supplementary material for: Antihypertensive drugs and pancreatic cancer risk in patients with chronic pancreatitis: a Danish nationwide population-based cohort study
Source: Br J Cancer. 2019 Sep 2;121(7):622–4. doi: 10.1038/s41416-019-0562-y (PMC6889405; doi:10.1038/s41416-019-0562-y)
Supplement: Supplementary file 1 — Supplementary Information [file 41416_2019_562_MOESM1_ESM.docx]

| **Online Supplementary Information. Baseline characteristics of 8,311 patients diagnosed with chronic pancreatitis in Denmark during 1996-2012, according to exposure to antihypertensive drugs at baseline.** | | | | | | | | |
| --- | --- | --- | --- | --- | --- | --- | --- | --- |
|  | | **Total**  **N (%)** | **ACEi**  **N (%)** | **ARB**  **N (%)** | **Aldosterone**  **N (%)** | **Beta**  **N (%)** | **Calcium**  **N (%)** | **Diuretics**  **N (%)** |
| **Total** | | **8,311** | **812** | **362** | **174** | **605** | **221** | **1,091** |
| **Median age, years (IQR)** | | **54 (45-64)** | **62 (53-71)** | **63 (54-70)** | **61 (51-66)** | **61 (52-70)** | **63 (54-73)** | **65 (55-74)** |
| **Age group** | |  |  |  |  |  |  |  |
|  | ≤45 years | 2,265 (27.3%) | 77 (9.5%) | 26 (7.2%) | 13 (7.5%) | 67 (11.1%) | 19 (8.6%) | 76 (7.0%) |
|  | 46-55 years | 2,366 (28.5%) | 181 (22.3%) | 78 (21.6%) | 51 (29.3%) | 153 (25.3%) | 47 (21.3%) | 208 (19.1%) |
|  | 56-65 years | 1,903 (22.9%) | 238 (29.3%) | 117 (32.3%) | 61 (35.1%) | 164 (27.1%) | 62 (28.1%) | 284 (26.0%) |
|  | >65 years | 1,777 (24.4%) | 316 (38.89%) | 141 (39.0%) | 49 (28.2%) | 221 (36.5%) | 93 (42.1%) | 523 (47.9%) |
| **Sex** | |  |  |  |  |  |  |  |
|  | Men | 5,498 (66.1%) | 556 (68.5%) | 217 (59.9%) | 103 (59.2%) | 382 (63.1%) | 131 (59.3%) | 548 (50.2%) |
|  | Women | 2,813 (33.9%) | 256 (31.5%) | 145 (40.1%) | 74 (40.8%) | 223 (36.9%) | 90 (40.7%) | 543 (49.8%) |
| **Socioeconomic status** | |  |  |  |  |  |  |  |
|  | Employed | 2,373 (28.6%) | 178 (21.9%) | 91 (25.1%) | 17 (9.8%) | 121 (20.0%) | 46 (20.8%) | 127 (11.6%) |
|  | Unemployed | 622 (7.5%) | 31 (3.8%) | >20 (-) | <10 (-) | 27 (4.5%) | <10 (-) | 32 (2.9%) |
|  | Retired | 5,027 (60.5%) | 590 (72.7%) | 244 (67.4%) | 150 (86.2%) | 447 (73.9%) | 164 (74.2%) | 920 (84.3%) |
|  | Other/unknown | 289 (3.5%) | 13 (1.6%) | <10 (-) | <10 (-) | 10 (1.7%) | <10 (-) | 12 (12.1%) |
| **Period of diagnosis** | |  |  |  |  |  |  |  |
|  | 1996-2003 | 3,973 (48.8%) | 240 (29.6%) | 74 (20.4%) | 69 (39.7%) | 203 (33.6%) | 118 (53.4%) | 441 (40.4%) |
|  | 2004-2012 | 4,338 (52.2%) | 572 (70.4%) | 288 (79.6%) | 105 (60.3%) | 402 (66.5%) | 103 (46.6%) | 650 (59.6%) |
| **Gagne Comorbidity Index** | |  |  |  |  |  |  |  |
|  | Low (score ≤ 0) | 4,394 (52.9%) | 404 (49.8%) | 217 (59.9%) | 32 (18.4%) | 263 (43.5%) | 104 (47.1%) | 442 (40.5%) |
|  | Moderate (score 1-2) | 3,088 (37.1%) | 266 (32.8%) | 99 (27.4%) | 91 (52.3%) | 222 (36.7%) | 77 (34.8%) | 408 (37.4%) |
|  | Severe (score >2) | 829 (10.0%) | 142 (17.5%) | 46 (12.7%) | 51 (29.3%) | 120 (19.8%) | 40 (18.1%) | 241 (22.1%) |
| **Comorbidity diagnoses** | |  |  |  |  |  |  |  |
|  | Alcohol-related disease | 3,543 (42.7%) | 246 (30.3%) | 106 (29.3%) | 114 (65.5%) | 224 (37.0%) | 59 (26.7%) | 371 (34.0%) |
|  | Smoking-related disease | 3,154 (38.0%) | 265 (32.6%) | 102 (28.2%) | 106 (60.9%) | 230 (38.0%) | 61 (27.6%) | 399 (36.7%) |
|  | Obesity | 277 (3.3%) | 56 (6.9%) | 30 (8.3%) | <10 (-) | 35 (5.8%) | 12 (5.4%) | 87 (8.0%) |
|  | Diabetes mellitus | 1,950 (23.5%) | 399 (49.1%) | 156 (43.1%) | 71 (40.8%) | 204 (33.7%) | 76 (34.4%) | 404 (37.0%) |
|  | Cerebrovascular disease | 696 (8.4%) | 134 (16.5%) | 49 (13.5%) | 28 (16.1%) | 107 (17.7%) | 36 (16.3%) | 181 (16.6%) |
|  | Cardiovascular disease | 1,151 (13.9%) | 256 (31.5%) | 99 (27.4%) | 47 (27.0%) | 239 (39.5%) | 89 (40.3%) | 328 (30.1%) |
|  | Liver disease | 1,362 (16.4%) | 100 (12.3%) | 43 (11.9%) | 93 (53.5%) | 104 (17.2%) | 25 (11.3%) | 232 (21.3%) |
|  | Renal disease | 392 (4.7%) | 91 (11.2%) | 35 (9.7%) | 13 (7.5%) | 73 (12.1%) | 25 (11.3%) | 139 (12.7%) |
|  | Hyperlipidemia | 511 (6.2%) | 120 (14.8%) | 60 (16.6%) | 16 (9.2%) | 101 (16.7%) | 28 (12.7%) | 135 (12.4%) |
|  | Previous malignancy | 585 (7.0%) | 63 (7.8%) | 41 (11.3%) | 10 (5.8%) | 57 (9.4%) | 29 (13.1%) | 103 (9.4%) |
|  | Ulcer | 1,197 (14.4%) | 108 (13.3%) | 55 (15.2%) | 39 (22.4%) | 103 (17.0%) | 39 (17.7%) | 200 (18.3%) |
|  | Chronic hepatitis B or C | 102 (1.2%) | <10 (-) | <10 (-) | <10 (-) | <10 (-) | <10 (-) | 11 (1.0%) |
| **Medication use at baseline** | |  |  |  |  |  |  |  |
|  | ACEi | 812 (9.8%) | 812 (100%) | 13 (4.4%) | 32 (18.4%) | 177 (29.3%) | 61 (27.6%) | 292 (26.8%) |
|  | ARB | 362 (4.4%) | 16 (2.0%) | 362 (100%) | <10 (-) | 74 (12.2%) | 25 (11.3%) | 122 (11.2%) |
|  | Aldosterone antagonist | 174 (2.1%) | 32 (3.9%) | <10 (-) | 174 (100%) | 31 (5.1%) | 6 (2.7%) | 112 (10.3%) |
|  | Beta-blocker | 605 (7.3%) | 177 (21.8%) | 74 (20.4%) | 31 (17.8%) | 605 (100%) | 37 (16.7%) | 233 (21.4%) |
|  | Calcium channel blocker | 221 (2.7%) | 61 (7.5%) | 25 (6.9%) | <10 (-) | 37 (6.1%) | 221 (100%) | 89 (8.2%) |
|  | Diuretics | 1,091 (13.1%) | 292 (36.0%) | 122 (33.7%) | 112 (64.4%) | 233 (38.5%) | 89 (40.3%) | 1,091 (100%) |

**ACEi**: Angiotensin-converting enzyme inhibitor; **ARB**: Angiotension-II receptor blockers; **Aldosterone**: Aldosterone receptor antagonists; **Beta**: Beta-blockers; **Calcium**: Calcium channel blockers; **IQR**: Inter-quartile range.
